# A study on the applicability of implantable microchip transponders for body temperature measurements in pigs

**DOI:** 10.1186/1751-0147-52-29

**Published:** 2010-05-05

**Authors:** Louise Lohse, Åse Uttenthal, Claes Enøe, Jens Nielsen

**Affiliations:** 1 National Veterinary Institute, Technical University of Denmark, Lindholm, DK-4771 Kalvehave, Denmark; 2 National Veterinary Institute, Technical University of Denmark, Bülowsvej 27, DK-1790 Copenhagen V, Denmark

## Abstract

**Background:**

The applicability of an electronic monitoring system using microchip transponders for measurement of body temperatures was tested in 6-week-old conventional Danish weaners infected with classical swine fever virus (CSFV). Subcutaneous tissue temperatures obtained by the implantable transponders were compared with rectal temperatures, recorded by a conventional digital thermometer.

**Methods:**

In a preliminary study, transponders were inserted subcutaneously at 6 different positions of the body of 5 pigs. The transponders positioned by the ear base provided the best correlation to rectal temperature. To test the stability of the monitoring system in a larger group of pigs, transponders were therefore inserted by the left ear base in a subsequent infection experiment with 30 pigs.

**Results:**

Generally, the microchip transponders measured a subcutaneous tissue temperature, which was about 1°C lower than the rectal temperature. However, a simple linear relationship between the measures of the two methods was found.

**Conclusions:**

Our study showed that the tested body monitoring system may represent a promising tool to obtain an approximate correlate of body temperatures in groups of pigs. In contrast, however, the tested system did not constitute a suitable tool to measure body temperatures of individual animals in the present pig infection experiment.

## Background

A major part of our research concerning viral infections in domestic animals involves investigations of the host-virus interaction based on infectious animal experimental studies [1-3]. The clinical monitoring of these studies inevitably includes registration of the animal's body temperature. Our standard procedure to obtain these data is to measure the rectal temperature using a digital thermometer, if necessary under restraint of the animal. Depending on the number of experimental pigs and the frequency of body temperature measurements required, the rectal recording method can be rather laborious and time consuming. Furthermore, the restraint of the animal may be stressful and compromise the well-being, leading to a hyperthermic response, although usually of short duration only [4]. In addition, the induced physical stress may increase the plasma cortisol concentration in the individual animal [4-6]. Since cortisol affects several physiological parameters e.g. blood cell profile and serum chemistry [6-8], stress-induced quantitative changes in the level of this hormone may bias experimental results.

To circumvent these problems, we are interested in alternative methods for body temperature monitoring in large animals using a minimal invasive technique, which in addition should be easy accessible, simple and fast in use. As our experimental models mostly involve the porcine species, we found it appropriate to test, whether a commercially available monitoring system would be applicable for body temperature measurements in pigs.

Pigs are increasingly used for biomedical research and advanced bio-telemetric equipment providing data of specific physiological variables, e.g. blood flow and pressure, ECG and body temperature, constitutes an opportunity for recording the body temperature without human interaction [9,10]. These systems, however, require surgical implantation for cardiac instrumentation.

A much more simple system using special ear tags with integrated sensors to measure the ear skin temperature in boars was tested by Bekkering and Hoy [11]. These authors, however, found that the skin temperature of the pig ear was not a reliable parameter for prediction of the rectal temperature, and as such, this method did not represent a reliable tool to monitor body temperature in our settings.

Thus, still looking for a system fulfilling our expectations, we have tested whether an electronic identification and body temperature monitoring technology presently applied in small experimental animals [12,13] could be transferred for use in pigs. This system is based on a radio-telemetric system using a programmable, injectable, microchip transponder with a built-in temperature sensor combined with a hand-held scanner for data collection. The system provides an opportunity to programme the transponder with further data, e.g. animal identification code, which can be linked to the temperature when recording. A few studies using this system have previously been performed in large animals, i.e. horses, goats, sheep [14] and pigs [15,16], with variable results.

In two animal experimental studies, already planned as parts of ongoing national research activities, we therefore wanted to compare microchip-based measurements of subcutaneous tissue temperature with digitally recorded rectal temperatures in pigs. Microchip transponders were placed subcutaneously in 35 pigs, which were either inoculated with classical swine fever virus (CSFV) of high or low virulence, respectively, or served as placebo-treated uninfected controls. We used a pig model infected with CSFV, a pathogen that may cause high fever, in order to test the system in healthy as well as febrile animals.

## Materials and methods

### Animals

In total, 35 six-week-old pigs, weight 10 - 16 kg, were obtained from a conventional Danish swine herd. At arrival, all pigs appeared healthy by clinical examination. All pigs were housed within the National Veterinary Institute's high containment experimental facilities and allowed to adapt to the new environment approximately one week before the start of the experiment. All pigs were fed once a day with commercial feed for weaning pigs and water was provided ad libitum. Straw was used for bedding. For experiment I, ambient temperature was not fixed and ranged within a temperature interval of 18-21°C. For experiment II, controlled heated air supply maintained a constant temperature of 20 ± 1°C throughout the experiment.

### Virus

The following strains of CSFV were used for inoculation in the experiments:

CSF0382: Koslov strain originating from the Czech Republic, characterized to be of high virulence (kindly supplied by the EU Community Reference Laboratory (CRL) for CSF, TiHo, Hannover), [17].

CSF0911: Glentorf strain originating from Germany, characterized to be of low virulence (kindly supplied by CRL, Hannover), [18].

CSF1019: Romania TM/120/07 field isolate obtained from domestic pigs, Romania 2007, characterized to be of medium/high virulence (kindly supplied by Dr Olaru, NRL, Romania and the CRL, Hannover).

All pigs were inoculated intranasally with a virus dose of 10^5 ^TCID_50_/pig.

### Electronic equipment

Bio Medic Data System (BMDS) (Plexx, the Netherlands): This system comprised implantable programmable temperature transponders (IPTT-300™) designed for non-surgical implantation into animals. The microchip transponder is a passive (battery free) cylinder shaped with a dimension of 2.2 × 14 mm placed in a needle assembly to be injected subcutaneously. Reading of the transponder is performed by a hand-held scanner (DAS 6007) to be oriented in line with the transponder within a distance of maximum 5 cm. Before insertion, transponders were programmed with the ID-number of the respective pig to avoid interference from neighbouring pigs during data collection.

Rectal temperatures were recorded with a digital thermometer, accuracy ± 0.1°C (Kruuse, Denmark).

In a laboratory set-up, the accuracy of transponders was tested against certified thermometers using a water bath representing 3 different temperatures, i.e. 37, 39 and 41°C. Accuracy of measurements for 4 transponders was found to be within the range of 0.1 - 0.4°C. In the animal experimental facilities, the repeatability of measurements of transponders within individual pigs was measured by 10 subsequent recordings in two randomly selected pigs. Repeatability was found to be 0.1 - 0.5°C.

### Experimental design

#### Experiment I

Five pigs were used. Microchip transponders were inserted at 6 different locations of each pig: T1+T2) Left and right ear base, respectively, below subcutis and associated connective and fat tissue, close to the cervical musculature. Left positioned transponder in vertical position, right positioned transponder in horizontal position. T3+T4) Ischio-rectal region. Left positioned transponder superficially inserted under the skin, right positioned transponder injected 1-2 cm into the depth of the ischio-rectal fossa in cranio-caudal direction, parallel to rectum. T5) Inguinal region, left side, transponder injected below subcutis and associated connective tissue, into the fascia close to the oblique abdominal musculature. T6) Neck region, transponder injected from a right lateral position 1-2 cm into the connective tissue, close to the neck musculature.

T1-T4 were inserted on post infection day (PID) -4, and T5+T6 were inserted PID 0. Before injection of a transponder, the skin area to be involved (10 × 10 cm) was shaved and surgically cleaned with 70% ethyl alcohol and 5% iodine, respectively.

On PID 0, all pigs were inoculated with CSFV-Koslov. Blood samples were collected on PID 0, 1, 3 and 7 for haematological and virological examinations.

Body temperature was recorded once a day with both telemetric equipment and digital thermometer. Readings for rectal temperature and microchip transponders, T1-T4, were obtained from start (PID -4) and followed during the entire experiment, while data for microchip transponders T5 and T6 were obtained from PID 0 and then followed to the end of the experimental period at PID 7, where all pigs were euthanized.

#### Experiment II

Thirty pigs were used. One microchip transponder was inserted vertically, deep subcutaneously at left ear base (position T1). Preparation procedure of the skin was the same as in experiment I. Pigs were divided into 3 groups of 10 individuals. On PID 0, the 3 groups were treated as follows: Group 1) control group, pigs mock-inoculated intranasally with Eagle's minimal essential medium (EMEM). Group 2) infected group, pigs inoculated with CSFV-Glentorf. Group 3) infected group, pigs inoculated with CSFV-Romania. Blood samples were collected on PID 0-7, 10, 15, 21/22 for further laboratory examination. Pigs were sequentially euthanized and autopsied. The experiment was terminated on PID 22.

All experimental procedures and animal manage protocols were carried out in accordance with the requirements of the Danish Animal Experimentation Inspectorate, license no. 2003/561-742.

### Virological examination

For both experiments, serum samples from all pigs collected at the day of euthanasia were tested for antibodies against CSFV by the virus neutralization assay previously described [19].

Quantitative real time RT-PCR for detection of CSFV RNA was performed on all serum samples from all pigs of the 2 experiments according to the protocol previously described by [20].

Virus isolation and detection by immunoperoxidase staining was performed on serum samples and/or organs of all pigs in this study by standard procedure of the institute according to Uttenthal *et al*. [20].

### Statistics

For experiment II, we adopted the approach proposed by Bland and Altman [21] to assess the limits of agreement between two methods of clinical measurement. Additionally, data were analysed to examine if there was a simple linear relationship between the digitally taken rectal temperature and the microchip-based measurement of subcutaneous tissue temperature in the pigs. Data was analysed by linear regression models using proc mixed in SAS^®^. We assumed that the population values of the dependent variable temp_T _(transponder temperature) was normally distributed for each value of the explanatory variable temp_R _(rectal temperature). This was evaluated by residual plots (not shown). In the analyses, possible effects from treatment group, day in study and a non-linear relationship between the two measures were taken into account. To adjust for the repeated measures, individual pigs were included as random effects in the models.

## Results

In experiment I, the implantation of the transponders did not provoke any adverse reactions, i.e. neither changes in behaviour, appetite, body temperature nor local tissue reaction were observed in any of the pigs. In experiment II, adverse reaction was found in one of 30 pigs. Thus, the transponder in pig 12 could not be recognized by the scanner after PID 5. In addition, the post mortem examination revealed a firm swelling at the injection site of this pig, indicating the development of a local reaction towards the implant or the surgical procedure. Temperature data from pig 12 were excluded after PID 5 when the pig was euthanized according to the experimental set-up. During the six days' observation period for this animal, the body temperature remained at a consistent level within the normal range, when measured by digital thermometer. The transponder generated data, however, revealed that from PID 1, temperatures remained up to 1.9°C lower for pig 12 compared to the readings obtained for other pigs in the same experimental group for the same period. This observation most likely reflected changed tissue conditions in pig 12 as a result of local inflammation. During the post mortem examination, transponders from 34 pigs could easily be recovered from the insertion sites, whereas the transponder in pig 12 could not be found.

In both experiment I and II, CSF virus, RNA and/or a neutralizing antibody response against CSFV were found in all virus-inoculated pigs, demonstrating all pigs to be infected.

### Experiment I

In the first part of the experiment (PID -4 - 0, i.e. before virus inoculation), when body temperatures of the pigs still were considered to be within normal level, rectal temperatures ranged from 39.2 to 40.5°C with a mean of 39.6°C (Standard deviation (SD) = 0.3). In comparison, subcutaneous temperatures, displayed by the 4 different transponders, showed lower values and were scattered over a much wider temperature interval, depending on transponder position (Table 1). The ear base positioned transponders T1) and T2) showed readings closest to the rectal temperatures, displaying differences around 1°C. Transponder data from the ischio-rectal region, T3) and T4), showed much larger deviations from rectal temperatures, with differences of more than 2.0°C.

**Table 1:** Rectal and subcutaneous temperatures (°C) on post infection days -4 to 0, experiment I

Reading location	Temperature range	Mean	SD	Mean difference (Rectal -- T_x_)
Rectal	39.2-40.5	39.6	0.3	-
T1	37.5-39.6	38.6	0.5	1.0
T2	37.2-39.4	38.4	0.6	1.2
T3	33.7-38.1	36.5	1.1	3.1
T4	35.8-38.8	37.2	0.3	2.4

T1) = transponder positioned at left ear base

T2) = transponder positioned at right ear base

T3) = transponder positioned at left ischio-rectal region

T4) = transponder positioned at right ischio-rectal region

**Figure 1:**
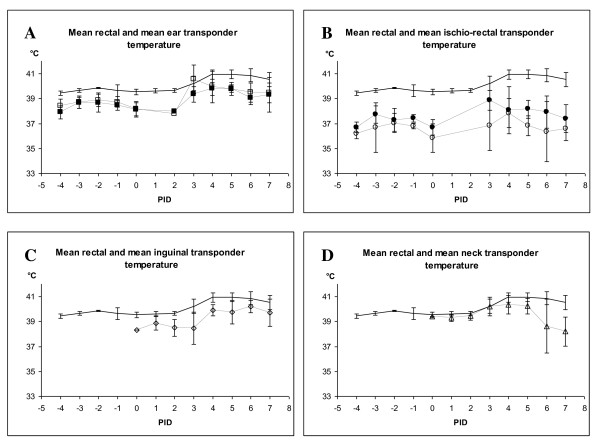
**A-D - Mean rectal and mean subcutaneous temperatures for experiment I**. Each dot represents the average ± SD for all pigs recorded on the specific reading the individual day. - rectal temperature, -white square- left and --black square- right ear transponder, -white circle- left and --black circle- right ischio-rectal transponder, -white triangle- neck transponder, -white diamond- inguinal transponder.

In the second part of the experiment (PID 1-7, i.e. after virus inoculation), the pigs developed pyrexia and rectal temperatures ranged from 39.4 to 41.6°C. Subcutaneous temperatures, displayed by 6 different transponders, showed a larger variation than observed in the first part and ranged from 34.0 to 42.9°C. Mean values for rectal and transponder temperatures have not been computed for this period, as body temperatures vary on a daily basis for individual pigs going through a course of swine fever, depending on the stage of infection for the individual animal. Therefore, all transponder data were compared with rectal temperatures through mean values for the five pigs on individual days and graphically visualized (Figure 1A-D). Before calculation of mean values, the data for the different transponder positions within individual pigs were compared (data not shown). The variation within each pig correlated well to the variation of the mean values for the corresponding sampling points from all five pigs. On this basis, we decided to proceed in the following infection experiment with the microchip transponder placed at the ear base position. This priority was made due to 1) consistency in difference between rectal standard and ear base positioned transponder, and 2) the convenience connected to the insertion and reading procedure of a transponder in this position.

### Experiment II

**Figure 2:**
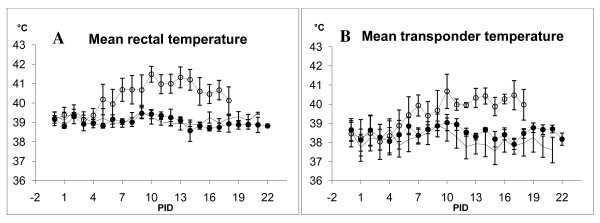
**A-B - Mean rectal and mean transponder temperatures for experiment II**. Each dot represents the average ± SD for all pigs in the respective groups recorded on the specific reading the individual day. - group 1 (control), --black circle-- group 2 (CSFV-Glentorf), --white circle-- group 3 (CFSV-Romania).

In experiment II, subcutaneous and rectal temperatures from the 3 different treatment groups were recorded and compared. Pigs in group 3 (CSFV-Romania) developed fevers (i.e. rectal temperature ≥ 40.0°C according to Eriksen [22]) on PID 5 and mean rectal temperature on individual days for this group stayed above normal level for the rest of the experimental period. For pigs in group 1) (control), and group 2) (CSFV-Glentorf), the rectal temperature remained at a temperature interval considered to be within normal physiological range (i.e. rectal temperature 38.5-40.0°C according to Eriksen [22]) for the entire experimental period. The development of body temperature over time within the 3 groups was concurrently registered by transponders as well as digital thermometers. The mean body temperature obtained for the pigs on individual days in each of the 3 groups, are shown in Figures 2A (rectal) and 2B (transponder), respectively. Unfortunately, standard deviation of the mean values of temperatures obtained by transponder measurements was larger compared to rectal recordings for all 3 groups, indicating less accuracy for the electronic monitoring system when used on individual animal level (table 2).

**Table 2:** Rectal and transponder temperatures (°C), experiment II

Group ID	N^a^	Temperature range			
		**rectal**	**max SD**	**transponder**	**max SD**

Group 1	10	38.9-39.6	0.4	37.6-38.9	0.8
Group 2	10	38.7-39.5	0.6	37.9-39.0	0.8
Group 3	10	39.2-41.5	0.8	38.0-40.7	1.0

Group 1: control group

Group 2: CSFV-Glentorf infected

Group 3: CSFV-Romania infected

N^a^: number of pigs per group. Note that it is the initial number of pigs, i.e. the number at post infection day 0 that is stated. According to the experimental set-up, pigs were sequentially euthanized, i.e. 3 pigs from each group in week 1 and 2, respectively, thus in the 3rd week, only 4 pigs in each group were left and killed by termination of the study.

The mean  of the differences (temp_R_-temp_T_) was  = 0.7819 and the standard deviation *s *of the differences was *s *= 0.6062. The limits of agreement was [-1.9701; 0.4063], thus with 95% confidence the temp_T _was within the range of 0.40°C above to 1.97°C below the temp_R_.

Simple linear relationship was found between temp_R _and temp_T _in a linear regression model including individual pig as random effects.

The linear relationship can be expressed as:

where the transponder temperature (temp_T_) had a statistically significant relationship with the rectal temperature (temp_R_) (*P *< 0.0001). There was no statistical significant effect of treatment group on the linear relationship.

In a model where the difference of the two measures in individual pigs was used as dependent variable, there was no statistical significant effect of treatment group, day in study or rectal temperature.

A sensitivity analysis was carried out for possible influence from pig 25 (group 3, CSFV-Romania), because the differences in transponder and rectal temperatures were relatively large (≥ 2.6) for all days in the study. Five out of the 6 largest differences in the experiment was observed (2.6; 2.7; 3.3; 3.3; 4.2) for pig 25 and already on PID 0 the difference was 2.1 indicating that there was a systematically large difference in the measurements. Pig 25 was euthanized and censored from the study on PID 6.

The sensitivity analysis showed that pig 25 significantly influenced the fit of the model. By the following necropsy of pig 25, the microchip transponder injected in this pig was found to be located within fatty tissue and not as aimed in the interface between subcutaneous tissue and cervical musculature. This post mortem finding may explain the diverging readings displayed by this specific transponder. For this reason, it was decided to delete pig 25 from further analyses.

**Figure 3:**
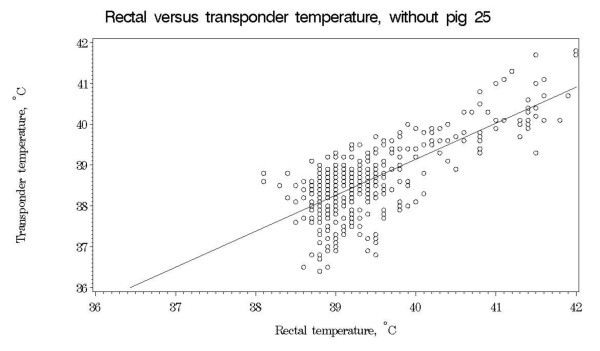
**Regression line for observations in experiment II**.

Omitting pig 25, however, only changed the parameter estimates slightly:

Using this regression equation, it can be calculated that a rectal temperature of e.g. 40°C corresponds to an expected mean transponder temperature of 39.1°C. Figure 3 shows the regression line for the data without pig 25. Residual plots were generated to confirm that the underlying assumptions for linear regression were justified (plots not shown).

## Discussion

In order to develop our system for body temperature recording in experimental pig studies, we have tested an electronic monitoring system based on subcutaneous insertion of microchip transponders. The system has been developed for use in small laboratory animals and produce reliable readings in marmosets [12] and rodents [13]. The microchip transponders have been tested in these animals, subcutaneously as well as intraperitoneally showing no significant difference from the rectal standard. As an alternative to rectal probes, the system is described as an easy, reliable and non-surgical implantable technology which provides further advantages including 1) animal welfare perspectives, i.e. reduction of stress associated with handling and restraint and 2) refinement of humane end-point criteria in experimental settings using continual measurements of body temperature as a supplement to life/death criteria. Immediately, the system therefore seemed to constitute an attractive tool for body temperature measurements to be challenged in our experimental pig animal model.

Transfer of this technology to large animals may face some practical challenges since the transponder is designed to be injected into tissue of much smaller animals with a reading distance between transponder and scanner for data collection of a maximum of 5 cm. Furthermore, the needle assembly is not produced with reference for the skin composition of a pig and only for subcutaneous injection. These constrains have to be taken into account, e.g. monitoring necessitates the operator of the scanner to be close to the individual pig of each recording. Regarding the subcutaneous transponder position, there may be a risk that the measured transponder temperature does not only reflect the true difference between the temperature of the rectal and subcutaneous tissue, respectively, but does include variation of ambient room temperature within the experimental unit.

Our continued interest in the above described system, however, depended on the level of agreement between the two different methods for measuring body temperature, i.e. whether the subcutaneous tissue temperature measured by transponder technology could be used as a correlate for the rectal temperature obtained by conventional digital thermometer recording. Therefore, we started out by challenging the technology through insertion of microchip transponders at different parts of the body of the pig: The chosen positions were suggested with regard to 1) body temperature levels expected to be comparable to rectal temperatures, 2) easiness of insertion of transponder, and 3) easily accessible readings.

The results generated in experiment I showed that position of the microchip transponder in the pig is highly critical with regard to temperature level and temperature consistency.

Five (T1-T5) of six microchip transponder readings paralleled the rectal readings (Figure 1A-C), however, the deviation from the rectal standard differed with position. Ear region and inguinal region data showed a temperature interval around 1°C below rectal measurements, respectively, while ischio-rectal transponders showed a difference of more than 2°C. This temperature difference is in contrast to the results published by Dunney *et al*. [15], who obtained very satisfactory results with transponders in the ichio-rectal position with readings closely following the rectal temperature. Dunney *et al*. [15] compared the same telemetric system as used in our study with the rectal standard by measurements of ten pigs of undefined age in ten days as a part of a larger clinical trial. Since only results of two representative pigs are displayed, it is not possible to compare actual calculated differences with our results. In a more recent study, the success from Dunney *et al*. [15] could not be repeated. Thus, Hartinger *et al*. [16] tested the system in mice, guinea pigs, rabbits and pigs. This study reported sufficient reliability of data in rabbits, only. When Hartinger *et al*. [16] injected the transponders subcutaneously just below ear-base position in 12 conventional cross-bred pigs, the temperature sensitive transponders provided neither reliability nor consistency in body temperature measurements of this species.

The T6 transponder (neck position) showed a reading pattern diverging from those of the T1-T5 positions (Figure 1D). In the pre-infection period, this transponder showed consistently close (almost identical) readings to the corresponding rectal readings. However, when the pigs developed fever due to the virus infection, the T6 transponder produced discrepant readings, not at all in line with the contemporary rectal reading. This observation could reflect a changed vasomotor action in this area of the body as a result of the systemic pyrexia in the virus-infected pigs.

In experiment II, the ear-positioned transponder provided tissue temperature readings, which paralleled the rectal readings. In accordance with the results of the first study, however, the measured transponder temperature level was lower (Figures 2A and 2B). The limits of agreement between the two measures were relatively wide. This may partly be explained by the limited number of paired readings in experiment II, especially at the end of the experimental period. Statistical evaluation of these data showed a simple linear relationship between the measures of the rectal and the transponder temperature. The results also showed that a minor proportion (1.5%) of animals with a rectal temperature above 40°C, which is considered as pyrexia, will not be detected by using the corresponding threshold of 39.1°C derived from the regression equation for the transponder temperature.

At termination of both experiments, the recovery of all but one transponder from the injection sites revealed that migration of the transponder apparently was not a problem.

Considering the applicability of the tested technology, the transponder position requires specific focus as this parameter seems to influence the temperature span between obtained transponder temperatures and rectal temperatures. In addition, an optimised insertion procedure is of importance since tissue reactions such as inflammation and haemorrhage may alter local tissue conditions, thus resulting in disturbed transponder temperature readings. Finally, it may be assumed that transponders inserted deeply into skeletal musculature may provide a better correlation to rectal recordings as such a position is likely to be protected by the influence of the ambient temperature. The reading limits of 5 cm together with the fact that the insertion device does not facilitate intramuscular injection rule out the possibility to use the tested system for intramuscular use in pigs.

## Conclusion

Addressing the limitations given by the size of the present data, the results indicate that the tested transponder monitoring system may constitute a practical tool to obtain a correlate of rectal temperatures in groups of pigs. Application of the technology could have potential interest for commercial production systems, where microchip-screening of body temperatures on a herd level would be very useful for early warning of changes in infection level, as also previously suggested for CSF [23]. Within an experimental setting, this system will benefit animal welfare, reduce experimental errors and ease practical procedures for recording of body temperature on a group level. In its present form, however, it is not suitable as a monitoring system of body temperature in individually sick pigs, where an exact body temperature can be a critically important parameter for further intervention procedures.

## Competing interests

The authors declare that they have no competing interests.

## Authors' contributions

LL researched background literature, participated in designing the study, carried out the practical aspects of the animal experiments, carried out data management, evaluating results and drafting the manuscript. AU supplied the funding of the study, participated in coordination and helped drafting the manuscript. CE carried out the statistical analysis and helped data management. JN conceived the idea, researched background literature, participated in designing the study, evaluating results and drafting the manuscript. All authors read and approved the final manuscript.
